# Infliximab reduces peripheral inflammation, neuroinflammation, and extracellular GABA in the cerebellum and improves learning and motor coordination in rats with hepatic encephalopathy

**DOI:** 10.1186/s12974-016-0710-8

**Published:** 2016-09-13

**Authors:** Sherry Dadsetan, Tiziano Balzano, Jerónimo Forteza, Ana Agusti, Andrea Cabrera-Pastor, Lucas Taoro-Gonzalez, Vicente Hernandez-Rabaza, Belen Gomez-Gimenez, Nisrin ElMlili, Marta Llansola, Vicente Felipo

**Affiliations:** 1 Laboratorio de Neurobiología, Centro Investigación Príncipe Felipe de Valencia, Eduardo Primo Yufera, 3, 46012 Valencia, Spain; 2 Instituto Valenciano de Patología, Unidad Mixta de Patología Molecular, Centro de Investigación Príncipe Felipe/Universidad Católica de Valencia, Valencia, Spain

**Keywords:** Hepatic encephalopathy, Neuroinflammation, Microglial activation, GABA, GAT-3, Infliximab, Neurological alterations

## Abstract

**Background:**

Peripheral inflammation contributes to the neurological alterations in hepatic encephalopathy (HE). Neuroinflammation and altered GABAergic neurotransmission mediate cognitive and motor alterations in rats with HE. It remains unclear (a) if neuroinflammation and neurological impairment in HE are a consequence of peripheral inflammation and (b) how neuroinflammation impairs GABAergic neurotransmission. The aims were to assess in rats with HE whether reducing peripheral inflammation with anti-TNF-α (1) prevents cognitive impairment and motor in-coordination, (2) normalizes neuroinflammation and extracellular GABA in the cerebellum and also (3) advances the understanding of mechanisms linking neuroinflammation and increased extracellular GABA.

**Methods:**

Rats with HE due to portacaval shunt (PCS) were treated with infliximab. Astrocytes and microglia activation and TNF-α and IL-1β were analyzed by immunohistochemistry. Membrane expression of the GABA transporters GAT-3 and GAT-1 was analyzed by cross-linking with BS3. Extracellular GABA was analyzed by microdialysis. Motor coordination was tested using the beam walking and learning ability using the Y maze task.

**Results:**

PCS rats show peripheral inflammation, activated astrocytes, and microglia and increased levels of TNF-α and IL-1β. Membrane expression of GAT-3 and extracellular GABA are increased, leading to impaired motor coordination and learning ability. Infliximab reduces peripheral inflammation, microglia, and astrocyte activation and neuroinflammation and normalizes GABAergic neurotransmission, motor coordination, and learning ability.

**Conclusions:**

Neuroinflammation is associated with altered GABAergic neurotransmission and increased GAT-3 membrane expression and extracellular GABA (a); peripheral inflammation is a main contributor to the impairment of motor coordination and of the ability to learn the Y maze task in PCS rats (b); and reducing peripheral inflammation using safe procedures could be a new therapeutic approach to improve cognitive and motor function in patients with HE (c).

## Background

Patients with liver cirrhosis may develop hepatic encephalopathy (HE) which begins with a non-evident phase called covert or minimal hepatic encephalopathy (MHE) in which the patients show motor in-coordination, psychomotor slowing, and mild cognitive impairment. These alterations may be detected by performing specific psychometric tests and affect quality of life of the patients [1]. There are no specific treatments for the neurological alterations in MHE.

Hyperammonemia and inflammation play synergistic roles in the induction of the cognitive and motor alterations in MHE, and there is a good correlation between the levels of inflammatory markers (IL-6, IL-18) in serum and the grade of MHE in cirrhotic patients [2–4]. Peripheral inflammation may lead to neuroinflammation [5] which, in turn, induces cognitive and motor impairment in different pathological situations including Alzheimer’s disease, multiple sclerosis, stroke, and aging [6–8]. PET studies show increased binding of TSPO ligands in the brain of cirrhotic patients with clinical HE [9] and in rats with HE due to bile duct ligation, suggesting the presence of neuroinflammation [10].

A main contributor to neuroinflammation in most pathological situations, including mild chronic hyperammonemia and HE, is microglia activation [11–16]. Microglia are the resident innate immune cells of the brain. Sustained activation of microglia to pro-inflammatory forms contributes to neurological alterations. However, microglia also plays neuroprotective roles via synaptic stripping, phagocytosis of debris and dysfunctional neurons, and neurogenesis promotion and may also produce anti-inflammatory cytokines [17].

Recent studies support the idea that peripheral inflammation may lead to cognitive and motor alterations in different pathological situations such as rheumatoid arthritis, diabetes, or after strong surgeries (reviewed in [18]). Patients with chronic inflammatory diseases are being treated with anti-TNF-α to reduce peripheral inflammation. It has been observed that this treatment improves cognitive function in patients with rheumatoid arthritis or sarcoidosis [19, 20]. Due to its large size, anti-TNF-α does not cross the blood–brain barrier, suggesting that its beneficial effects are a consequence of reduced peripheral inflammation.

We hypothesized that reducing peripheral inflammation could also improve cognitive and motor function in MHE. The first aim of this work was to assess whether peripheral treatment with anti-TNF-α prevents cognitive and motor impairment in rats with MHE. This would provide a new therapeutic approach to treat MHE. To reach this aim, we assessed whether chronic intravenous treatment with infliximab, an anti-TNF-α used in clinical practice, improves motor coordination and learning ability in rats with a portacaval shunt (PCS), a main model of MHE recommended by the International Society for Hepatic Encephalopathy [21].

The mechanisms responsible for motor and cognitive alterations in MHE are beginning to be clarified in animal models. Different types of cognitive and motor alterations are due to different mechanisms involving different brain areas [1]. Neuroinflammation in hippocampus impairs some types of learning and memory [22–24]. Rats with hyperammonemia and MHE show neuroinflammation in the hippocampus. Treatment of PCS rats with sildenafil or of hyperammonemic rats with sulforaphane reduces neuroinflammation in the hippocampus and improves spatial learning and memory [14, 15]. In hyperammonemic rats, increasing extracellular cGMP reduces some types of neuroinflammation and of learning and memory but not others [16]. These recent reports support that neuroinflammation plays an essential role in impairment of hippocampus-associated learning and memory processes in hyperammonemia and MHE.

Both in cirrhotic patients and in rats with MHE, the cerebellum is selectively affected at the earliest stages. Non-invasive blood flow measurement in the cerebellum detects cerebral alterations in cirrhotic patients earlier than psychometric tests. Blood flow was selectively increased in the cerebellum in cirrhotic patients with MHE and, more slightly but also significantly, in cirrhotic patients who were classified as “without MHE” according to the psychometric hepatic encephalopathy score (PHES) [25]. Bimanual coordination which is mainly modulated by the cerebellum [26, 27] is also impaired in patients with or without MHE, correlating with increased blood flow in the cerebellum. This indicates that alterations in the cerebellum occur at very earliest stages of MHE, even before it can be detected by the PHES [25].

Also in rats with hyperammonemia or MHE cerebellum is affected at early stages. Rats with hyperammonemia and MHE due to portacaval shunts or bile duct ligation show neuroinflammation, which is more evident in the cerebellum than in other brain areas [11, 28], and increased extracellular GABA levels and GABAergic tone in the cerebellum [29, 30]. Both neuroinflammation and increased extracellular GABA in the cerebellum contribute to impair motor coordination and ability to learn a Y maze task. Treatments with anti-inflammatories such as ibuprofen or MAP kinase p38 inhibitors reduce neuroinflammation and improve motor function and the ability to learn the Y maze task [12, 28, 29]. Reducing GABA_A_ receptors activation also restores motor coordination and the ability to learn the Y maze task [29, 30]. This suggests that increased extracellular GABA in the cerebellum could mediate the effects of neuroinflammation on motor coordination and Y maze learning. We have recently shown that in hyperammonemic rats, treatment with sulforaphane reduces neuroinflammation and normalizes GABAergic neurotransmission in the cerebellum [13]. We hypothesized that increased peripheral inflammation would play a main role in the induction of neuroinflammation and associated alterations in GABAergic neurotransmission in the cerebellum of rats with MHE due to PCS.

A second main aim of this work was to assess whether reducing peripheral inflammation reduces neuroinflammation and extracellular GABA in the cerebellum of PCS rats. As a tool to reduce peripheral inflammation, we used anti-TNF-α. To assess if neuroinflammation is a consequence of peripheral inflammation, we treated rats with anti-TNF-α before performing the PCS surgery, to prevent the associated inflammation and assess whether this reduces neuroinflammation. Finally, a last aim was to advance in the understanding of the mechanisms linking neuroinflammation and increased extracellular GABA in the cerebellum in MHE.

## Methods

**Fig. 1:**
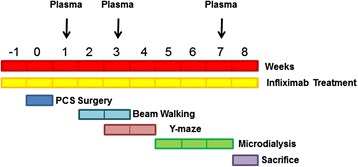
Scheme of the experimental design

*Portacaval anastomosis and treatment with infliximab*. Male Wistar rats (220–240 g) were subjected to end-to-side portacaval shunt (PCS) as described by Lee and Fisher [31]. Control rats were sham operated, and the vein was clamped for 10 min. The experiments were approved by the Comite de Experimentación y Bienestar Animal (CEBA) of our center and performed in accordance with guidelines of the Directive of the European Commission (2010/63/EU) for care and management of experimental animals. Rats were randomly distributed into four groups: sham; sham + infliximab; PCS; PCS + infliximab. Infliximab (Remicade; Merck Sharp & Dohme, Spain) was dissolved in water and administered by i.v. injection (5 mg/kg) in the tail vein as in [32]. The first administration of infliximab was performed 2 days before PCS surgery. Weekly treatment with infliximab was maintained until the sacrifice except during behavioral tests, when infliximab was administered every 2 weeks. Control rats were injected i.v. with saline. The experimental design is summarized in Fig. 1. A total of four experiments were performed, using eight rats per group in each experiment. Rats from each group and experiment were randomly divided into three subgroups for studies of microdialysis, membrane expression, or perfusion for immunohistochemistry studies. The number of rats used for each parameter is indicated in the corresponding figure legend.

*Determination of PGE2, IL-6, IL-10, and IL-4 in plasma.* Plasma samples were collected from tail vein at weeks 1, 3, and 7 after PCS surgery and stored at −80 °C. Prostaglandin E2 (PGE2) was measured using the ELISA Biotrak system (Amersham Bioscience, UK). IL-6, IL-10, and IL-4 levels were analyzed by western blot. Samples were subjected to electrophoresis and immunoblotting using primary antibodies against IL-10 and IL-4 (1:1000) from Abcam (ab9969 and ab9811, respectively) and IL-6 (1:500) from BioSource (ARC0062). Secondary antibodies were anti-rabbit (1:4000) IgG conjugated with alkaline phosphatase. The images were captured using the Hewlett Packard ScanJet 5300C, and band intensities were quantified using the AlphaImager 2200 program.

*Ammonia determination in blood*. Blood (20 μL) was taken from the tail vein. Blood ammonia was measured immediately after blood collection with the Ammonia Test Kit II for the PocketChemBA system (Arkay, Inc., Kyoto, Japan).

*Learning of a conditional discrimination task in a Y maze*. Learning ability was tested as in [33] in a wooden Y-shaped maze. Rats must learn where the food is depending on the color of the walls. Rats performed 10 trials per day, until the completion of a criterion of 10 correct responses in the same day or a maximum of 250 trials.

*Motor coordination and beam walking test*. Motor coordination was tested using the beam walking test in a wood strip (20-mm diameter) as described by Gonzalez-Usano et al. [30] Rats have to cross a 1-m long wooden stick located approximately 1 m above the ground and two observers count the number of slips committed by the rats. The number of foot faults (slips) is recorded as a measure of in-coordination.

*In vivo microdialysis*. Rats were anesthetized using isoflurane and a microdialysis guide was implanted in cerebellum (AP −10.2, ML −1.6, and DV −1.2), as in [34]. After 48 h, a microdialysis probe was implanted in the freely moving rat. Probes were perfused (3 μl/min) with artificial cerebrospinal fluid (in mM): NaCl, 145; KCl, 3.0; CaCl_2_, 2.26; buffered at pH 7.4 with 2 mM phosphate. After a 2–3-h stabilization period, samples were collected every 30 min. EDTA was added to the samples at a final concentration of 4 mM and stored at −80 °C until analysis of cGMP and GABA levels.

*GABA determination*. To assess the basal level of extracellular GABA in the cerebellum, eight microdialysis samples were collected. GABA concentration was measured by HPLC as described by Canales et al. [35].

*Membrane surface expression of GAT-1 and GAT-3 transporters by cross-linking with BS*_*3*_*in cerebellar slices*. It was analyzed as described by Boudreau and Wolf [36]. Rats were sacrificed by decapitation and their brains were transferred into ice-cold Krebs buffer (in mmol/L): NaCl 119, KCl 2.5, KH_2_PO_4_ 1, NaHCO_3_ 26.2, CaCl_2_ 2.5, and glucose 11, aerated with 95 % O_2_ and 5 % CO_2_ at pH 7.4. Cerebellum was dissected and transversal slices (400 μm) were obtained using a vibrotome. Slices were added to tubes containing ice-cold Krebs buffer with or without 2 mM BS_3_ (Pierce, Rockford, IL) and incubated for 30 min at 4 °C. Cross-linking was terminated by adding 100-mM glycine (10 min, 4 °C). The slices were homogenized by sonication for 20 s. Samples treated with or without BS_3_ were analyzed by western blot using anti-GAT-3 or anti-GAT-1 (1:1000; Abcam, ab431 and ab426, respectively; Cambridge, UK). The surface expression of transporters was calculated as the difference between the intensity of the bands without BS3 (total protein) and with BS3 (non-membrane protein).

*Brain immunohistochemistry*. At week 8 after PCS surgery, the rats were anesthetized with sodium pentobarbital and transcardially perfused with 0.9 % saline followed by 4 % paraformaldehyde in 0.1-M phosphate buffer (pH 7.4). Brains were removed and post-fixed in the same fixative solution for 24 h at 4 °C. Then, the samples were placed inside histology cassettes and processed for permanent paraffin embedding on a Leica ASP 300 tissue processor (Leica Microsystems). The processor performed the following steps: 60 min in formalin, 45 min in 70 % ethanol, 45 min in 90 % ethanol, four changes in 100 % ethanol (one for 45 min and three for 60 min, respectively), three changes in xylene (45, 60, and 75 min, respectively), and three changes in paraffin (Histowax, melting point 56–58 °C) for 60 min. Five-micrometer-thick, paraffin-embedded sections (5 μm) were cut and mounted on coated slide glass. The tissue sections were then processed with the Envision Flex + kit (DAKO) blocking endogenous peroxidase activity for 5 min and then incubating with primary antibody. The reaction was visualized by Envision Flex + horseradish peroxidase for 20 min and finally diaminobenzidine for 10 min. Sections were counterstained with Mayer’s hematoxylin (DAKO S3309; ready to use) for 5 min.

The primary antibodies used were anti-Iba-1 (Wako, 019-19741; 1:300 for 30 min), anti-GFAP (DAKO, IR524; ready to use for 20 min), anti-TNF-α (Abcam, ab66579; 1:2000 for 45 min), anti-IL-1β (Abcam, ab9722, 1:100 for 30 min), anti-GAT3 (Abcam, ab431; 1:500 for 40 min), and anti-GAT-1 (Abcam, ab426; 1:200 for 40 min).

*Immunohistochemical quantification*. It was performed using ImageJ (1.48v). For analysis of microglial activation and of the areas stained by GFAP or GAT-3 antibodies, the area of interest was selected. Using Auto Local Threshold and analyzed particle functions, the intensity thresholds and size filter were applied.

To measure the area and perimeter of microglia, the Bernsen method was used and 2000–20,000 size filter was applied. For each rat, at least 30–40 cells were quantified. The result was expressed as percentage respect to control.

For GFAP or GAT-3 staining, no size filter was applied. For each rat, at least 10 fields (×56 for GFAP and ×40 for GAT-3) were quantified. The result was expressed as percentage of control rats.

TNF-α and IL-1β positive cells were manually counted using ImageJ. For each rat, at least 10 fields (×40) were quantified and results were expressed as percentage of control rats.

To quantify the intensity of GAT1, Purkinje neurons were manually outlined using ROI manager function and the selection was measured. Mean gray value for each Purkinje cell was measured. For each rat, at least 80–100 cells were quantified.

*Statistical analysis*. Results are expressed as mean ± SEM. Data were analyzed by one-way analysis of variance (ANOVA) followed by Tukey’s post hoc test. For the statistical analysis of ammonia levels in blood and peripheral inflammation two-way ANOVA with repeated measures, followed by Bonferroni post hoc test was used. Two-way ANOVA with repeated measures, followed by Bonferroni post hoc test, was also used for the analysis of learning index in the Y maze test. *p* < 0.05 is considered to indicate statistically significant differences.

## Results

### Infliximab reduces peripheral inflammation and neuroinflammation in the cerebellum of PCS rats

PCS rats show peripheral inflammation, with increased plasma levels of pro-inflammatory cytokine IL-6 and of PGE-2 and reduced levels of anti-inflammatory cytokine IL-10.

One week after surgery, PGE-2 levels were increased (*p* < 0.05) in PCS rats to 2.8 ± 0.8 pg/μl compared to 0.8 ± 0.1 pg/μl in control (sham operated) rats. Treatment with infliximab reduced PGE-2 levels in PCS rats to 1.0 ± 0.1 pg/μl.

A similar effect was observed for pro-inflammatory IL-6 which increased (*p* < 0.01) in plasma of PCS rats to 154 ± 17 % of control rats and was normalized by treatment with infliximab to 112 ± 8 % of controls.

The anti-inflammatory IL-10 was reduced (*p* < 0.01) in plasma of PCS rats to 58 ± 8 % of control rats and was normalized by treatment with infliximab to 80 ± 10 % of controls. No significant effects were observed on IL-4.

PCS rats show increased (*p* < 0.0001) blood ammonia levels. At 7 weeks, the levels were 202 ± 55 μM while in control (sham) rats were 43 ± 11 μM. Treatment with infliximab did not affect ammonia levels in sham or PCS rats. Ammonia levels in PCS rats treated with infliximab were 244 ± 30 μM.

**Fig. 2:**
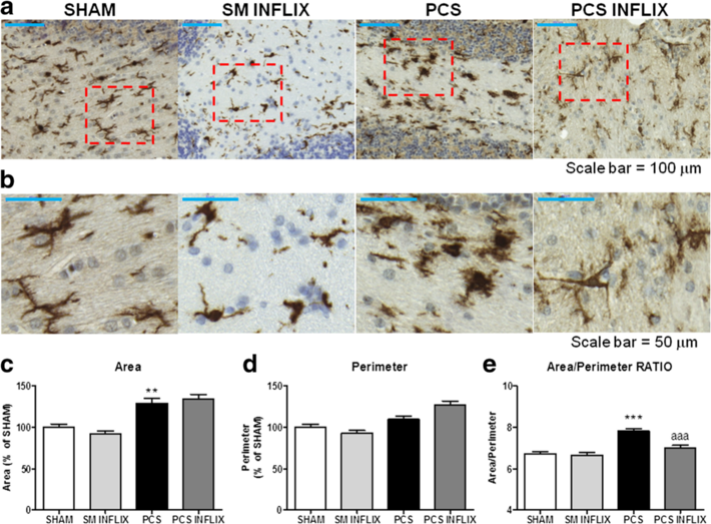
Infliximab reduces microglial activation in the white matter of the cerebellum of PCS rats. Immunohistochemistry was performed as indicated in the “Methods” section using antibody against Iba-1. Representative low (×20; **a**) and high (×56; **b**) magnification images are shown. Perimeter (**c**), area (**d**), and ratio area/perimeter (**e**) of microglial cells were quantified in the white matter of the cerebellum. Values are the mean ± SEM of 4 rats per group. Values significantly different from controls are indicated by *asterisks* and from PCS rats by *a*. **p < 0.01; ***p < =0.005; *aaa p* < 0.005. *Scale bar* low magnification (**a**) = 100 μm; *scale bar* high magnification = 50 μm

PCS rats show activation of microglia in the white matter of cerebellum as revealed the morphology of microglia immunostained with anti-Iba-1 (Fig. 2). The ratio area/perimeter of microglia, a measure of the grade of activation, was increased (*p* < 0.001) in PCS rats to 7.8 ± 0.1 compared to control rats (6.7 ± 0.1). In PCS rats treated with infliximab, microglial activation is strongly reduced and their ratio area/perimeter of microglia was 7.0 ± 0.1, significantly lower (*p* < 0.0001) than in PCS rats and not different from controls.

No activation of microglia was found in the molecular layer. The ratio area/perimeter of microglia was even slightly reduced (*p* < 0.05) in PCS rats to 5.5 ± 0.1 compared to control rats (6.0 ± 0.1). In PCS rats treated with infliximab, the ratio area/perimeter of microglia was 5.6 ± 0.1, not different from control or PCS rats (not shown).

**Fig. 3:**
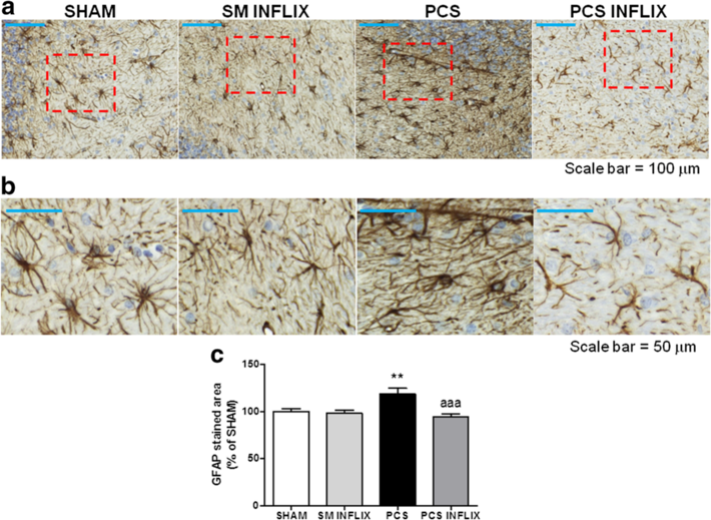
Infliximab reduces astrocyte activation in the white matter of the cerebellum of PCS rats. Immunohistochemistry was performed using antibody against GFAP. Representative low (×20; **a**) and high (×56; **b**) magnification images are shown. The area stained by GFAP antibody was quantified (**c**) in the white matter of the cerebellum. Values are the mean ± SEM of 4 rats per group. Values significantly different from controls are indicated by *asterisks* and from PCS rats by *a*. **p < 0.01; *aaa p* < 0.005. *Scale bar* low magnification (**a**) = 100 μm; *scale bar* high magnification = 50 μm

PCS rats also show fibrous astrocyte activation in the white matter of cerebellum, as clearly shown in Fig. 3 by its morphology when stained with GFAP. The content of GFAP in the astrocytes (calculated as the area covered by GFAP) in the white matter of the cerebellum of PCS rats was increased to 119 ± 6 % of controls (*p* < 0.01). Treatment with infliximab normalized the activation and morphology of astrocytes in PCS rats (Fig. 3) and GFAP amount (95 ± 3 % of controls).

**Fig. 4:**
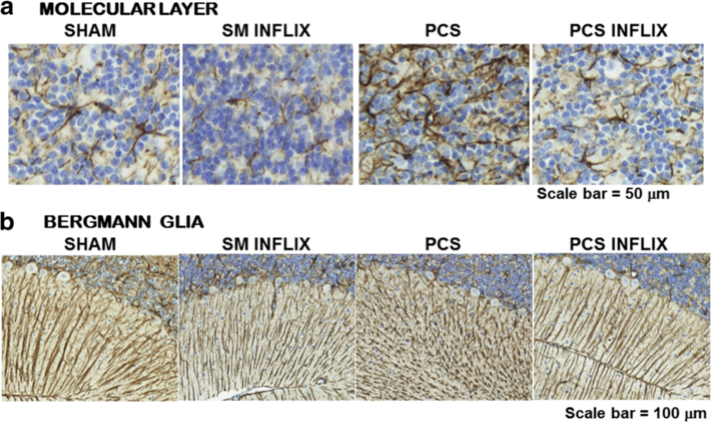
Infliximab reduces astrocyte activation in the molecular layer and prevents damage of Bergmann glia in the cerebellum of PCS rats. Immunohistochemistry was performed using antibody against GFAP. Representative images are shown. *Scale bar* magnification (**a**) = 50 μm; (**b**) = 100 μm

A similar effect was found in the granular layer. The GFAP content of PCS rats was increased to 121 ± 9 % of controls (*p* < 0.001). Treatment with infliximab normalized GFAP amount in PCS rats to 95 ± 10 % of controls (Fig. 4a).

We also analyzed the effects on Bergmann glia, a subtype of cerebellar astrocytes that reside next to Purkinje neurons. When stained with GFAP, Bergmann glial fibers present a disorganized and hypertrophied morphology in PCS rats compared to control rats while in PCS rats treated with infliximab show intact morphology (Fig. 4b).

**Fig. 5:**
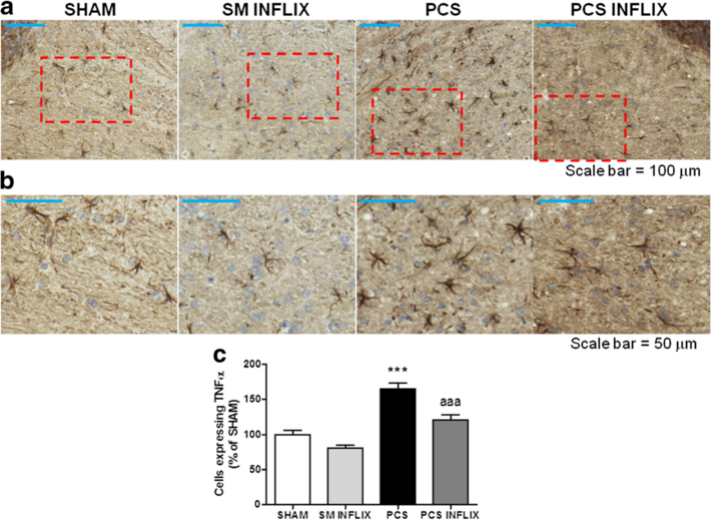
Infliximab reduces the number of TNF-α positive cells in the white matter of the cerebellum of PCS rats. Immunohistochemistry was performed using antibody against TNF-α. Representative low (×20; **a**) and high (×40; **b**) magnification images are shown. The number of TNF-α positive cells was quantified (**c**) in the white matter of the cerebellum. Values significantly different from controls are indicated by *asterisks* and from PCS rats by *a*. ***p < =0.005; *aaa p* < 0.005. *Scale bar* low magnification (**a**) = 100 μm; *Scale bar* high Magnification = 50 μm

PCS rats also showed increased levels of the pro-inflammatory markers TNF-α and IL-1β in the cerebellum. For TNF-α, this can be clearly seen in the immunostaining shown in Fig. 5. Quantification of the immunostaining shows that in cerebellum of PCS rats, the number of cells expressing TNF-α increases (*p* < 0.001) in the cerebellum to 165 ± 9 % of controls. Treatment with infliximab normalizes the immunostaining and the number of cells expressing TNF-α (120 ± 9 % of controls) in PCS rats (Fig. 5).

**Fig. 6:**
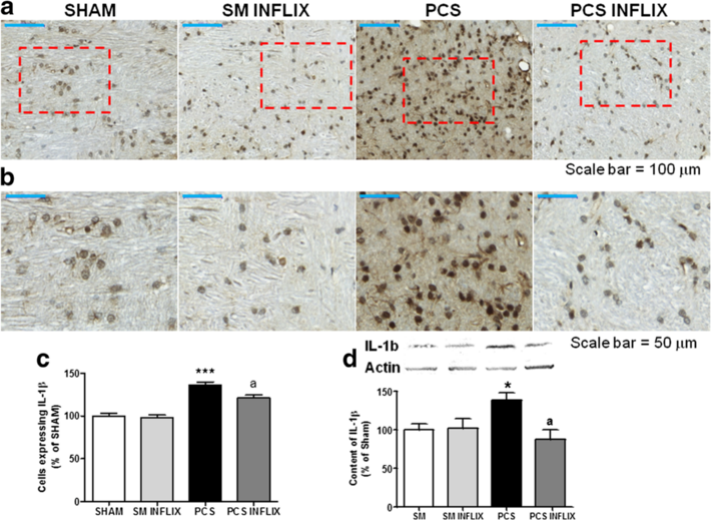
Infliximab reduces the number of IL-1β positive cells and the content of IL-b in white matter of the cerebellum of PCS rats. Immunohistochemistry was performed using antibody against IL-1β. Representative low (×20; **a**) and high (×40; **b**) magnification images are shown. The number of IL-1β positive cells was quantified (**c**) in the white matter of the cerebellum. Values are the mean ± SEM of 4 rats per group. The content of IL-1β in homogenates of the whole the cerebellum was also analyzed by western blot (**d**). Representative images are shown. Values significantly different from controls are indicated by *asterisks* and from PCS rats by *a*. *p < =0.05; ***p < =0.005; *a p* < 0.05. *Scale bar* low magnification (**a**) = 100 μm; *scale bar* high magnification = 50 μm

The same occurs for Il-1β as shown in the immunostaining shown in Fig. 6. The number of cells expressing IL-1β in the cerebellum increases in PCS rats to 136 ± 4 % of controls (*p* < 0.001). Infliximab normalizes the immunostaining and the number of cells expressing IL-1β (119 ± 4 % of controls) in PCS rats (Fig. 6c). The content of IL-1β in the cerebellum, quantified by western blot, was increased in PCS rats to 138 ± 10 % of controls (*p* < 0.05) and was normalized to 87 ± 13 % of controls by infliximab treatment (Fig. 6d).

### Altered membrane expression of GABA transporters and extracellular GABA in the cerebellum of PCS rats; effects of infliximab

**Fig. 7:**
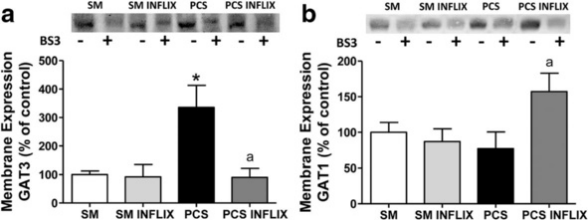
Altered membrane expression of GABA transporters in the cerebellum of PCS rats. Membrane expression of GAT3 (**a**) and GAT1 (**b**) was analyzed using the BS3 crosslinker procedure as described in the “Methods” section. Samples, incubated in the absence or presence of BS3, were subjected to western blotting using antibodies against each transporter. Representative images are shown. Samples in the absence of BS3 represent the total amount of each protein. Samples in the presence of BS3 represent the non-membrane fraction. The intensities of the bands were quantified and membrane expression was calculated as the difference of intensity between samples without and with BS3. Values are expressed as percentage of control rats and are the mean ± SEM of 8 rats per group for GAT1; 6 sham rats treated with vehicle and 7 sham rats treated with infliximab; and 6 PCS rats treated with vehicle and 5 PCS rats treated with infliximab for GAT3. Values significantly different from control rats are indicated by asterisks and from PCS rats by *a*. * *p* < 0.05; *a p* < 0.05

The membrane expression of the GABA transporter GAT-3 is strongly increased in PCS rats to 336 ± 77 % of controls (*p* < 0.001). Treatment with infliximab completely eliminated this increase in PCS rats. Membrane expression of GAT-3 returned to 90 ± 32 % of controls (Fig. 7a).

Concerning the GAT-1 transporter, its membrane expression is not altered in PCS rats, remaining at 77 ± 24 % of controls (Fig. 7b). However, treatment with infliximab strongly increased GAT-1 in membranes of PCS rats (157 ± 26 % of controls, *p* < 0.05) but not of control rats (87 ± 18 % of controls) (Fig. 7b).

**Fig. 8:**
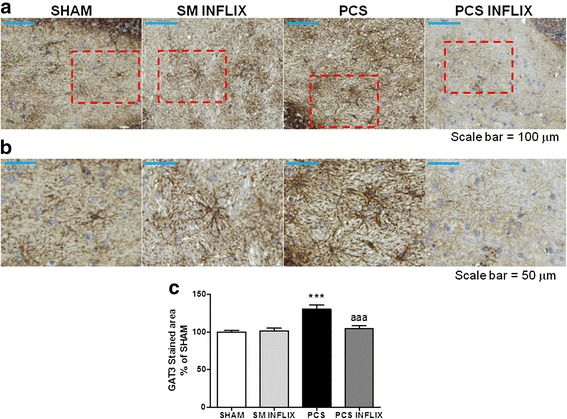
Expression of GAT3 in activated astrocytes in the white matter of the cerebellum. Immunohistochemistry was performed using antibody against GAT3. Representative low (×20; **a**) and high (×40; **b**) magnification images are shown. The *ara* stained by the GAT3 antibody was quantified (**c**) in the white matter of the cerebellum. Values are the mean ± SEM of 4 rats per group. Values significantly different from controls are indicated by *asterisks* and from PCS rats by *a*. ***p < =0.005; *aaa p* < 0.005. *Scale bar* low magnification (**a**) = 100 μm; *scale bar* high magnification = 50 μm

We also analyzed by immunohistochemistry the expression of GAT-3 and GAT-1 in the cerebellum. As shown in Fig. 8, GAT-3 is mainly expressed in astrocytes and is especially increased in activated astrocytes. The intensity of GAT-3 immunostaining (Fig. 8c) was increased (*p* < 0.001) in the cerebellum of PCS rats to 131 ± 5 % of controls and was normalized by treatment with infliximab (105 ± 4 % of controls).

**Fig. 9:**
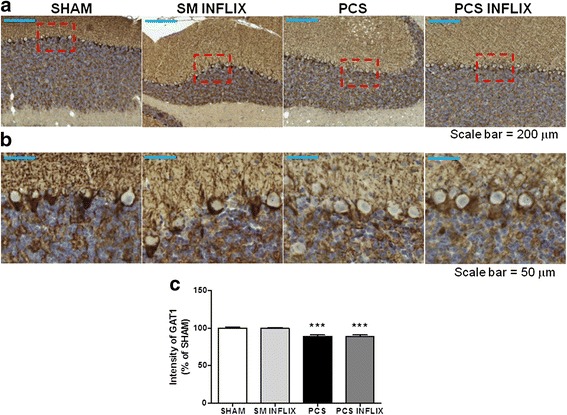
Expression of GAT1 in Purkinje neurons. Immunohistochemistry was performed using antibody against GAT1. Representative low (×10; **a**) and high (×40; **b**) magnification images are shown. The intensity of GAT1 staining was quantified (**c**) in Purkinje neurons. Values are the mean ± SEM of 4 rats per group. Values significantly different from controls are indicated by *asterisks*. ***p < =0.005. *Scale bar* low magnification (**a**) = 200 μm; *scale bar* high magnification = 50 μm

GAT-1 is expressed in the granular layer and surrounding Purkinje cells (Fig. 9). The intensity of GAT-1 immunostaining (Fig. 9c) was slightly reduced (*p* < 0.01) around Purkinje cells in PCS rats to 89 ± 2 % of controls and was not affected by treatment with infliximab (90 ± 3 % % of controls).

**Fig. 10:**
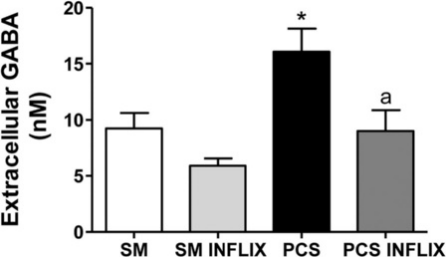
Infliximab normalizes extracellular GABA in the cerebellum of PCS rats. Control (sham) and PCS rats treated with vehicle or infliximab (inflix) were subjected to in vivo microdialysis in the cerebellum. Extracellular GABA was measured by HPLC in the eight initial samples of each rat. Values are the mean ± SEM of 12 sham rats treated with vehicle and 15 sham rats treated with infliximab; and 13 PCS rats treated with vehicle and 12 PCS rats treated with infliximab. Values significantly different from control are indicated by *asterisks* and from PCS rats by *a*. * *p* < 0.05; ** *p* < 0.01; *a p* < 0.05

The extracellular concentration of GABA was analyzed in the cerebellum of freely moving rats by microdialysis. Extracellular GABA was increased in PCS rats to 182 ± 22 % of controls (*p* < 0.05). Treatment with infliximab completely eliminated this increase in PCS rats. Extracellular GABA returned to 98 ± 19 % of controls (Fig. 10).

### Infliximab restores motor coordination and the ability to learn the Y maze task in PCS rats

**Fig. 11:**
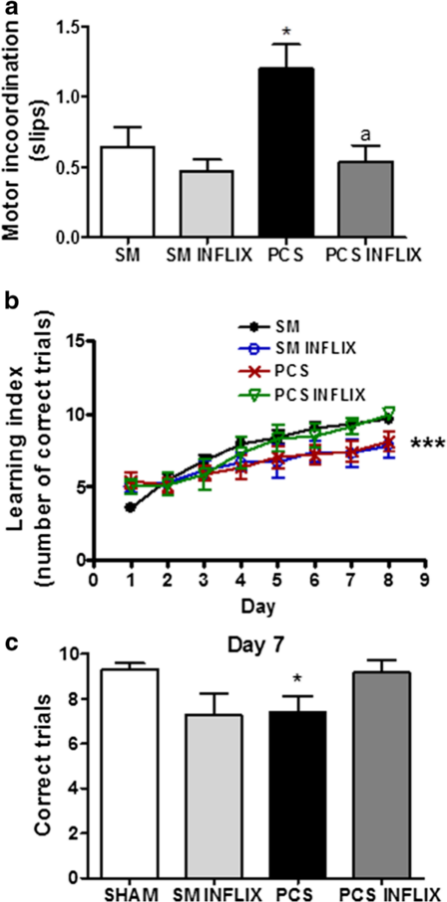
Infliximab restores motor coordination and the ability to learn the Y maze task in PCS rats. Control (sham) and PCS rats treated with vehicle or infliximab (inflix) were subjected to the beam walking (**a**) test and the conditional discrimination learning test in the Y maze (**b**, **c**). **b** shows the number of correct trails on each day and **c** on day 7. Values are the mean ± SEM of 22 sham rats treated with vehicle, sham rats treated with infliximab, 25 PCS rats treated with vehicle and 23 PCS rats treated with infliximab in the beam walking and 7 sham rats treated with vehicle, 7 sham rats treated with infliximab, 6 PCS rats treated with vehicle and 6 PCS rats treated with infliximab in the Y maze task. Two-way ANOVA with repeated measures, followed by Bonferroni post hoc test, was used for statistical analysis in **b**, variables being group treatment and training day. Values significantly different from control are indicated by *asterisks* and from PCS rats by *a*. * *p* < 0.05; *** *p* < 0.0001; *a p* < 0.05

PCS rats show motor in-coordination in the beam walking tests (Fig. 11a), with increased number of slips (1.2 ± 0.2, *p* < 0.001) compared to control rats (0.6 ± 0.2 slips). Treatment with infliximab completely normalized the motor coordination in PCS rats (0.5 ± 0.1 slips).

PCS rats also show reduced ability to learn the Y maze task (Fig. 11b, c). As shown in Fig. 7b, the learning index improved with days of training in all groups. The ANOVA analysis shows that the learning index was lower in PCS than in control rats (*p* < 0.001; *F* = 7.587). PCS rats treated with infliximab completely recover learning ability which was similar to controls (Fig. 11b).

The data for day 7 are shown in Fig. 11c. Control rats perform correctly 9.3 ± 0.3 trials while PCS rats perform significantly less (*p* = 0.01) correct trails (7.4 ± 0.7). PCS rats treated with infliximab recover learning ability and perform correctly 9.2 ± 0.5 trials, similar to control rats.

## Discussion

The results reported provide two main advances in the understanding of the mechanisms leading to neurological alterations in rats with HE due to PCS: (1) impairment of motor coordination and of the ability to learn the Y maze task is induced by peripheral inflammation and may be prevented by reducing it and (2) neuroinflammation leads to altered neurotransmission in the cerebellum of PCS rats by increasing membrane expression of the GABA transporter GAT-3 and extracellular GABA.

**Fig. 12:**
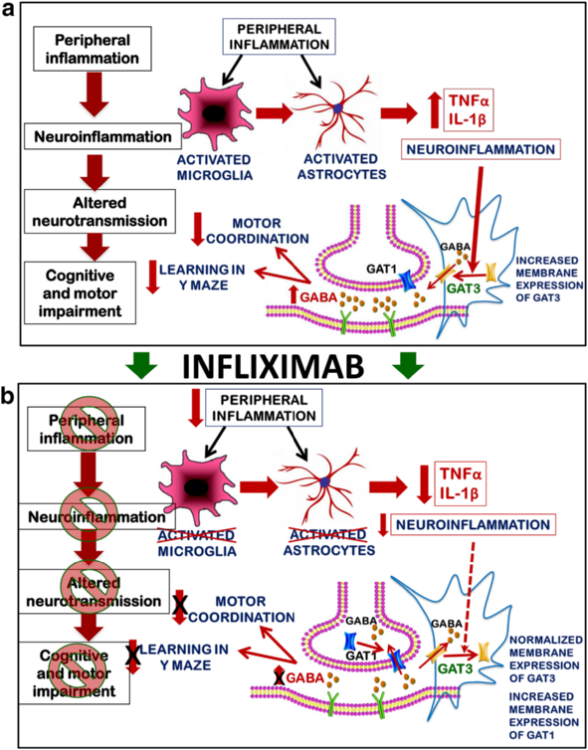
Proposed model for the mechanisms involved in the impairment of motor coordination and learning and memory in rats with HE and for their improvement by infliximab. **a** Peripheral inflammation in PCS rats induces activation of microglia and astrocytes in the cerebellum leading to increased expression of the pro-inflammatory markers TNF-α and IL-1β. This neuroinflammation leads to enhanced membrane expression of GAT-3, a GABA transporter that in activated astrocytes reverses the transport direction, resulting in a release of GABA to the extracellular fluid leading to increased extracellular GABA in the cerebellum. The increase in GABA leads to motor in-coordination and to reduced ability to learn the Y maze task. **b** Treatment with infliximab reduces the peripheral inflammation which prevents the activation of microglia and astrocytes and the associated enhancement of TNF-α and IL-1β expression in the cerebellum. This leads to normalized membrane expression of GAT3 in the astrocytes while it increases the expression of GAT1 in Purkinje neurons facilitating GABA uptake from the extracellular space resulting in normalized extracellular GABA concentration. As a consequence, the motor coordination is improved and the learning ability in the Y maze is restored

On the bases of the results obtained, we propose in Fig. 12 a putative model for the possible mechanisms involved in the impairment of motor coordination and learning and memory in rats with HE and for their improvement by infliximab.

The results show that peripheral inflammation plays a main role in the cognitive and motor alterations in rats with MHE. Peripheral inflammation leads to neuroinflammation, which would alter GABAergic neurotransmission resulting in impaired motor coordination and ability to learn the Y maze task. We found strong astrocyte and microglial activation in the white matter, as occurs in other pathological situations such as spinocerebellar ataxia type 1, in which neuronal dysfunction, in the absence of neurodegeneration, induces glial activation [37]. We also found damage of Bergmann glia, which is next to Purkinje cells, and may contribute to alter their function and GABAergic neurotransmission.

Treatment with infliximab reduces peripheral inflammation but not ammonia levels. The lack of effect on ammonia levels indicates that the beneficial effects of infliximab in PCS rats are not due to hepatoprotection. Reducing peripheral inflammation with infliximab is associated with reduced microglia and astrocyte activation and levels of pro-inflammatory TNF-α and IL-1β. Infliximab-induced reduction of neuroinflammation is associated with normalization of GABAergic neurotransmission and of motor coordination and the ability to learn the Y maze task. This suggests that peripheral inflammation is a main contributor to the impairment of motor coordination and of the ability to learn the Y maze task in PCS rats.

These results also suggest that treatments with anti-TNF-α could be a new therapeutic approach to improve cognitive and motor function in patients with MHE or clinical HE. Anti-TNF-α is already being used in clinical practice to reduce peripheral inflammation in the treatment of chronic inflammatory diseases. It has been observed that this treatment improves cognitive function in patients with rheumatoid arthritis or sarcoidosis [19, 20] and also in patients with Alzheimer’s disease [38, 39]. However, it should be taken into account that a possible problem of anti-TNF-α treatment in some patients is that it may induce liver injury [40, 41].

In any case, the results reported here provide experimental evidence that peripheral inflammation is a main contributor to neuroinflammation in rats with HE due to PCS and support that reducing inflammation by safe procedures would reduce neuroinflammation and improve cognitive and motor function in cirrhotic patients with minimal HE. The results reported show that neuroinflammation and the associated neurological alterations are reversible at early stages of MHE. It is unclear if the effects would be also reversible after long periods of neuroinflammation. More sustained neuroinflammation may trigger a series of changes in the brain which may lead to structural alterations, including in the worse cases neuronal degeneration, which can make some of the alterations irreversible. However, taking into account that most neurological alterations in patients with liver cirrhosis and HE are reversible after liver transplantation [42], it would be expected that treatment with anti-TNF-α could also restore most of these neurological alterations.

There is increasing evidence that many chronic diseases and other situations associated with chronic inflammation (e.g., strong surgeries) result in mild cognitive and motor impairment. As proposed in Fig. 12, the process by which peripheral inflammation leads to these neurological alterations would involve induction of neuroinflammation in different brain areas, including the cerebellum. Neuroinflammation would induce alterations in neurotransmission which would be responsible for the cognitive and motor alterations.

There are several mechanisms by which peripheral inflammation may be transduced to the brain to induce neuroinflammation. Cytokines are large peptides that do not readily cross the blood–brain barrier, [43]; however, one known signaling pathway is through active transport of certain cytokines allowing their entry into the brain parenchyma [44]. Furthermore, blood cytokines may activate their receptors in endothelial cells and trigger the release of inflammatory factors into the brain. For example, in rats injected with LPS, blood IL-6 activates its receptors in endothelial cells leading to activation of STAT3 which increases cyclooxygenase 2 and PGE2 in the cerebral cortex [45]. Immune-to-brain signaling through activation of vagal afferent nerves has also been reported as has direct entry of cytokines at circumventricular regions (e.g., the organum vasculosum lateralis terminalis) due to the lack of an intact blood–brain barrier in these brain areas [46]. In stronger inflammation cases, infiltration of immune cells from the periphery is pivotal for exacerbation of the pathology [47–49].

In PCS rats, we have not seen the infiltration of immune cells. We have observed that, at early phases after PCS surgery, IL-1β accumulates especially around blood vessels, suggesting that activation of interleukin receptors in endothelial cells may be contributing to neuroinflammation induction.

Further studies to identify the mechanisms by which peripheral inflammation induces neuroinflammation in HE would allow identifying additional targets to improve cognitive and motor function. The present data, together with the above reports on the utility of anti-TNF-α to improve cognition in different pathologies, suggest that it is worth trying it also in MHE and in clinical HE.

Another relevant contribution of this work is that we also identify for the first time molecular mechanisms by which neuroinflammation alters GABAergic neurotransmission in the cerebellum of PCS rats in vivo and how treatment with infliximab restores it. It is shown that, in rats with HE, the amount and membrane expression of the GAT3 transporter of GABA is increased in activated astrocytes. Under normal conditions, GAT-3 transports GABA from the extracellular space into astrocytes. However, under pathological conditions, this transport is reversed and GAT-3 releases GABA from astrocytes to the extracellular fluid. Wu et al. [49] showed that in a mouse model of Alzheimer’s disease, activated astrocytes in the hippocampus release GABA through GAT-3 transporters. Blocking GAT-3 reduced GABA concentration and GABA currents, while blocking GAT-1 transporters enhanced GABA currents, supporting that, under these pathological conditions, GAT-3 is releasing while GAT-1 is taking up GABA [34]. A similar GAT-3-mediated release of GABA would occur in activated astrocytes in the cerebellum of hyperammonemic rats and likely also in other pathological situations associated with neuroinflammation in the cerebellum.

It has already been shown that increased GABAergic tone in the cerebellum impairs motor coordination [50]. Also, mice lacking the GABA transporter subtype 1 (GAT1) show increased extracellular GABA and reduced motor coordination [51]. Moreover, rats developmentally exposed to polychlorinated biphenyls show motor in-coordination which correlates with extracellular GABA levels in the cerebellum [52].

The neuroinflammation-induced increase of extracellular GABA in the cerebellum would therefore be responsible for motor in-coordination in rats with HE due to PCS. Restoration of motor coordination in PCS rats by infliximab would be due to the normalization of extracellular GABA levels.

Increased extracellular GABA in the cerebellum would be also responsible for impairment of the glutamate-NO-cGMP pathway which, in turn, would result in reduced ability to learn the Y maze task. This has been already shown in PCS and in hyperammonemic rats. Reducing activation of GABA_A_ receptors with bicuculline, pregnenolone sulfate or GR3027 reduces GABAergic tone and restores the function of the pathway and the ability to learn the Y maze task [29, 30, 53]. The normalization of extracellular GABA in the cerebellum of PCS rats by infliximab would be therefore responsible for the normalization of the function of the glutamate-NO-cGMP pathway and of learning in the Y maze.

In this work, infliximab treatment started 2 days before PCS surgery. This was done to assess if neuroinflammation is a consequence of peripheral inflammation. If infliximab treatment starts once inflammation is already present, it is likely that the process inducing neuroinflammation could have already started making it more difficult to discern the contribution of peripheral inflammation. However, this experimental design is not the best one to assess the therapeutic utility of infliximab to reverse the cognitive and motor alterations in patients or models of MHE. This would require that treatment begins after the cognitive and motor alterations are already present. We have recently shown that treating PCS or hyperammonemic rats with sulforaphane, sildenafil, or extracellular cGMP, starting the treatments after establishment of the neurological impairment, reduces neuroinflammation in hippocampus and improves some types of spatial learning and memory [14–16], supporting the possible therapeutic utility of these approaches. On the other hand, treatment with anti-TNF-α compounds improves cognitive function in patients with rheumatoid arthritis or sarcoidosis [19, 20], indicating that it may be also beneficial if administered after establishment of cognitive impairment. This suggests that anti-TNF-α treatment could also be beneficial in patients with MHE or clinical HE. The above reports show that neuroinflammation in hyperammonemia and MHE may be reduced by different treatments acting on different initial targets. All of them improve cognitive and motor function in animal models. Further studies to assess which treatment has less secondary effects or more beneficial effects or the possible use of a combination therapy may led to new promising treatments for MHE and HE.

## Conclusions

In summary, the results reported show that, in PCS rats, peripheral inflammation leads to neuroinflammation in the cerebellum, which increases GAT-3 expression in membrane and extracellular GABA, altering GABAergic neurotransmission and resulting in impaired motor coordination and ability to learn the Y maze task. Infliximab reduces peripheral inflammation, microglia and astrocyte activation, and neuroinflammation and normalizes GAT-3 membrane expression, extracellular GABA and GABAergic neurotransmission, motor coordination, and the ability to learn the Y maze task. This supports that

- neuroinflammation is associated with altered GABAergic neurotransmission and increased GAT-3 membrane expression and extracellular GABA;
- peripheral inflammation is a main contributor to the impairment of motor coordination and of the ability to learn the Y maze task in PCS rats;
- reducing peripheral inflammation using safe procedures (anti-TNF-α or other) could be a new therapeutic approach to improve cognitive and motor function in patients with MHE or clinical HE.

## Abbreviations

CEBA: Comite de Experimentación y Bienestar Animal
HE: Hepatic encephalopathy
MHE: Minimal hepatic encephalopathy
PCS: Portacaval shunt
PGE2: Prostaglandin 2
PHES: Psychometric hepatic encephalopathy score

## References

[1] Felipo V (2013). Hepatic encephalopathy: effects of liver failure on brain function. Nat Rev Neurosci.

[2] Shawcross DL, Davies NA, Williams R, Jalan R (2004). Systemic inflammatory response exacerbates the neuropsychological effects of induced hyperammonemia in cirrhosis. J Hepatol.

[3] Montoliu C, Piedrafita B, Serra MA, del Olmo JA, Urios A, Rodrigo JM, Felipo V (2009). IL-6 and IL-18 in blood may discriminate cirrhotic patients with and without minimal hepatic encephalopathy. J Clin Gastroenterol.

[4] Felipo V, Urios A, Montesinos E, Molina I, El Mlili N, Garcia-Torres ML, Civera M, del Olmo JA, Ortega J, Martinez-Valls J, Serra MA, Cassinello N, Wassel A, Rodrigo JM, Jordá E, Montoliu C (2012). Contribution of hyperammonemia and inflammatory factors to cognitive impairment in minimal hepatic encephalopathy. Metab Brain Dis.

[5] Biesmans S, Meert TF, Bouwknecht JA, Acton PD, Davoodi N, De Haes P, Kuijlaars J, Langlois X, Matthews LJ, Ver Donck L, Hellings N, Nuydens R (2013). Systemic immune activation leads to neuroinflammation and sickness behavior in mice. Mediators Inflamm.

[6] McGeer EG, McGeer PL (2010). Neuroinflammation in Alzheimer’s disease and mild cognitive impairment: a field in its infancy. J Alzheimers Dis.

[7] Janssen B, Vugts DJ, Funke U, Molenaar GT, Kruijer PS, van Berckel BN, Lammertsma AA, Windhorst AD (2016). Imaging of neuroinflammation in Alzheimer’s disease, multiple sclerosis and stroke: recent developments in positron emission tomography. Biochim Biophys Acta.

[8] Ownby RL (2010). Neuroinflammation and cognitive aging. Curr Psychiatry Rep.

[9] Cagnin A, Taylor-Robinson SD, Forton DM, Banati RB (2006). In vivo imaging of cerebral “peripheral benzodiazepine binding sites” in patients with hepatic encephalopathy. Gut.

[10] Kong X, Luo S, Wu JR, Wu S, De Cecco CN, Schoepf UJ, Spandorfer AJ, Wang CY, Tian Y, Chen HJ, Lu GM, Yang GF, Zhang LJ (2016). (18)F-DPA-714 PET imaging for detecting neuroinflammation in rats with chronic hepatic encephalopathy. Theranostics.

[11] Rodrigo R, Cauli O, Gomez-Pinedo U, Agusti A, Hernandez-Rabaza V, Garcia-Verdugo JM, Felipo V (2010). Hyperammonemia induces neuroinflammation that contributes to cognitive impairment in rats with hepatic encephalopathy. Gastroenterology.

[12] Agusti A, Cauli O, Rodrigo R, Llansola M, Hernández-Rabaza V, Felipo V (2011). p38 MAP kinase is a therapeutic target for hepatic encephalopathy in rats with portacaval shunts. Gut.

[13] Hernandez-Rabaza V, Cabrera-Pastor A, Taoro-Gonzalez L, Gonzalez-Usano A, Agusti A, Balzano T, Llansola M, Felipo V (2016). Neuroinflammation increases GABAergic tone and impairs cognitive and motor function in hyperammonemia by increasing GAT-3 membrane expression. Reversal by sulforaphane by promoting M2 polarization of microglia. J Neuroinflammation.

[14] Hernandez-Rabaza V, Agusti A, Cabrera-Pastor A, Fustero S, Delgado O, Taoro-Gonzalez L, Montoliu C, Llansola M, Felipo V (2015). Sildenafil reduces neuroinflammation and restores spatial learning in rats with hepatic encephalopathy: underlying mechanisms. J Neuroinflammation.

[15] Hernández-Rabaza V, Cabrera-Pastor A, Taoro-González L, Malaguarnera M, Agustí A, Llansola M, Felipo V (2016). Hyperammonemia induces glial activation, neuroinflammation and alters neurotransmitter receptors in hippocampus, impairing spatial learning: reversal by sulforaphane. J Neuroinflammation.

[16] Cabrera-Pastor A, Hernandez-Rabaza V, Taoro-Gonzalez L, Balzano T, Llansola M, Felipo V. In vivo administration of extracellular cGMP normalizes TNF-α and membrane expression of AMPA receptors in hippocampus and spatial reference memory but not IL-1β, NMDA receptors in membrane and working memory in hyperammonemic rats. Brain Behav Immun. 2016;57:360–70. doi:10.1016/j.bbi.2016.05.011. Epub 2016 May 14. PMID: 27189036. 10.1016/j.bbi.2016.05.011 27189036

[17] Chen Z, Trapp BD (2016). Microglia and neuroprotection. J Neurochem.

[18] Montoliu C, Llansola M, Felipo V (2015). Neuroinflammation and neurological alterations in chronic liver diseases. Neuroimmunology and Neuroinflammation.

[19] Raftery G, He J, Pearce R, Birchall D, Newton JL, Blamire AM, Isaacs JD (2012). Disease activity and cognition in rheumatoid arthritis: an open label pilot study. Arthritis Res Ther.

[20] Elfferich MD, Nelemans PJ, Ponds RW, De Vries J, Wijnen PA, Drent M (2010). Everyday cognitive failure in sarcoidosis: the prevalence and the effect of anti-TNF-alpha treatment. Respiration.

[21] Butterworth RF, Norenberg MD, Felipo V, Ferenci P, Albrecht J, Blei AT, Group Authors: ISHEN Commission Expt Models HE (2009). Experimental models of hepatic encephalopathy: ISHEN guidelines. Liver Int.

[22] Czerniawski J, Miyashita T, Lewandowski G, Guzowski JF (2015). Systemic lipopolysaccharide administration impairs retrieval of context-object discrimination, but not spatial, memory: Evidence for selective disruption of specific hippocampus-dependent memory functions during acute neuroinflammation. Brain Behav Immun.

[23] Wei P, Liu Q, Li D, Zheng Q, Zhou J, Li J (2015). Acute nicotine treatment attenuates lipopolysaccharide-induced cognitive dysfunction by increasing BDNF expression and inhibiting neuroinflammation in the rat hippocampus. Neurosci Lett.

[24] Cao XZ, Ma H, Wang JK, Liu F, Wu BY, Tian AY, Wang LL, Tan WF (2010). Postoperative cognitive deficits and neuroinflammation in the hippocampus triggered by surgical trauma are exacerbated in aged rats. Prog Neuropsychopharmacol Biol Psychiatry.

[25] Felipo V, Urios A, Giménez-Garzó C, Cauli O, Andrés-Costa MJ, González O, Serra MA, Sánchez-González J, Aliaga R, Giner-Durán R, Belloch V, Montoliu C (2014). Non invasive blood flow measurement in cerebellum detects minimal hepatic encephalopathy earlier than psychometric tests. World J Gastroenterol.

[26] Debaere F, Wenderoth N, Sunaert S, Van Hecke P, Swinnen SP (2004). Cerebellar and premotor function in bimanual coordination: parametric neural responses to spatiotemporal complexity and cycling frequency. Neuroimage.

[27] Pollok B, Butz M, Gross J, Schnitzler A (2007). Intercerebellar coupling contributes to bimanual coordination. J Cogn Neurosci.

[28] Cauli O, Rodrigo R, Piedrafita B, Boix J, Felipo V (2007). Inflammation and hepatic encephalopathy: ibuprofen restores learning ability in rats with porto-caval shunts. Hepatology.

[29] Cauli O, Mansouri MT, Agusti A, Felipo V (2009). Hyperammonemia increases GABAergic tone in cerebellum but decreases it in rat cortex. Gastroenterology.

[30] Gonzalez-Usano A, Cauli O, Agusti A, Felipo V (2014). Pregnenolone sulphate restores the glutamate-nitric oxide-cGMP pathway and extracellular GABA in cerebellum and learning and motor coordination in hyperammonemic rats. ACS Chem Neurosci.

[31] Lee SH, Fisher B (1961). Portocaval shunt in the rat. Surgery.

[32] Karson A, Dermitas T, Bayramgürler D (2013). Chronic administration of infliximab (TNF-α inhibitor) decreases depression and anxiety-like behaviour in rat model of chronic mild stress. Basic Clin Pharmacol Toxicol.

[33] Aguilar MA, Miñarro J, Felipo V (2000). Chronic moderate hyperammonemia impairs active and passive avoidance behavior and conditional discrimination learning in rats. Experimental Neurol.

[34] Monfort P, Corbalán R, Martinez L (2001). Altered content and modulation of soluble guanylate cyclase in the cerebellum of rats with portacaval anastomosis. Neuroscience.

[35] Canales JJ, Elayadi A, Errami M (2003). Chronic hyperammonemia alters motor and neurochemical responses to activation of group I metabotropic glutamate receptors in the nucleus accumbens in rats in vivo. Neurobiol Dis.

[36] Boudreau AC, Wolf ME (2005). Behavioral sensitization to cocaine is associated with increased AMPA receptor surface expression in the nucleus accumbens. J Neurosci.

[37] Cvetanovic M, Ingram M, Orr H, Opal P (2015). Early activation of microglia and astrocytes in mouse models of spinocerebellar ataxia type 1. Neuroscience.

[38] Tobinick EL, Gross H (2008). Rapid cognitive improvement in Alzheimer’s disease following perispinal etanercept administration. J Neuroinflammation.

[39] Cheng X, Shen Y, Li R (2014). Targeting TNF: a therapeutic strategy for Alzheimer’s disease. Drug Discov Today.

[40] Parekh R, Kaur N (2014). Liver injury secondary to anti-TNF-alpha therapy in inflammatory bowel disease: a case series and review of the literature. Case Rep Gastrointest Med.

[41] Ghabril M, Bonkovsky HL, Kum C, Davern T, Hayashi PH, Kleiner DE, Serrano J, Rochon J, Fontana RJ, Bonacini M; US Drug-Induced Liver Injury Network. Liver injury from tumor necrosis factor-α antagonists: analysis of thirty-four cases. Clin Gastroenterol Hepatol. 2013;11(5):558–564.e3 10.1016/j.cgh.2012.12.025 PMC3865702 23333219

[42] Atluri DK, Asgeri M, Mullen KD (2010). Reversibility of hepatic encephalopathy after liver transplantation. Metab Brain Dis.

[43] Quan N (2008). Immune-to-brain signaling: how important are the blood-brain-barrier-independent pathways. Mol Neurobiol.

[44] Banks WA, Kastin AJ, Broadwell RD (1995). Passage of cytokines across the blood-brain-barrier. Neuroimmunomodulation.

[45] Rummel C, Sachot C, Poole S, Luheshi GN (2006). Circulating interleukin-6 induces fever through a STAT3-linked activation of COX-2 in the brain. Am J Physiol Regul Integr Comp Physiol.

[46] D’Mello C, Le T, Swain MG (2009). Cerebral microglia recruit monocytes into the brain in response to tumor necrosis factor α signaling during peripheral organ inflammation. J Neurosci.

[47] Gimenez MA, Sim J, Archambault AS, Klein RS, Russell JH (2006). A tumor necrosis factor receptor 1 dependent conversation between central nervous system specific t cells and the central nervous system is required for inflammatory infiltration of the spinal cord. Am J Pathol.

[48] Blaževski J, Petković F, Momčilović M, Jevtić B, Mostarica Stojković M, Miljković D (2015). Tumor necrosis factor stimulates expression of CXCL12 in astrocytes. Immunobiology.

[49] Wu Z, Guo Z, Gearing M, Chen G (2014). Tonic inhibition in dentate gyrus impairs long-term potentiation and memory in an Alzheimer’s disease model. Nat Commun.

[50] Hanchar HJ, Dodson PD, Olsen RW, Otis TS, Wallner M (2005). Alcohol-induced motor impairment caused by increased extrasynaptic GABA(A) receptor activity. Nat Neurosci.

[51] Chiu CS, Brickley S, Jensen K, Southwell A, Mckinney S, Cull-Candy S (2005). GABA transporter deficiency causes tremor, ataxia, nervousness, and increased GABA-induced tonic conductance in cerebellum. J Neurosci.

[52] Boix J, Cauli O, Felipo V (2010). Developmental exposure to polychlorinated biphenyls 52, 138 or 180 affects differentially learning or motor coordination in adult rats. Mechanisms involved. Neuroscience.

[53] Johansson M, Agusti A, Llansola M, Montoliu C, Strömberg J, Malinina E (2015). GR3027 antagonizes GABA A receptor potentiating neurosteroids and restores spatial learning and motor coordination in rats with hepatic encephalopathy. Am J Physiol Gastrointest Liver Physiol.

